# In-hospital resource utilization in surgical and transcatheter aortic valve replacement

**DOI:** 10.1186/s12872-015-0118-x

**Published:** 2015-10-22

**Authors:** Jochen Reinöhl, Klaus Kaier, Anja Gutmann, Stefan Sorg, Constantin von zur Mühlen, Matthias Siepe, Hardy Baumbach, Martin Moser, Annette Geibel, Andreas Zirlik, Philipp Blanke, Werner Vach, Friedhelm Beyersdorf, Christoph Bode, Manfred Zehender

**Affiliations:** Department of Cardiology and Angiology I, Heart Center Freiburg University, Hugstetter Str. 55, 79106 Freiburg, Germany; Department of Cardiovascular Surgery, Heart Center Freiburg University, Freiburg, Germany; Department of Diagnostic Radiology, Medical Center-University of Freiburg, Freiburg, Germany; Department of Cardiovascular Surgery, Robert-Bosch-Krankenhaus Stuttgart, Stuttgart, Germany; Center for Medical Biometry and Medical Informatics, Medical Center-University of Freiburg, Freiburg, Germany

**Keywords:** Transcatheter aortic valve implantation, Aortic valve replacement, Resource utilization, Risk prediction, Cost, Length of stay, Total hospitalization since procedure

## Abstract

**Background:**

Little is known about preoperative predictors of resource utilization in the treatment of high-risk patients with severe symptomatic aortic valve stenosis. We report results from the prospective, medical-economic “TAVI Calculation of Costs Trial”.

**Methods:**

In-hospital resource utilization was evaluated in 110 elderly patients (age ≥ 75 years) treated either with transfemoral (TF) or transapical (TA) transcatheter aortic valve implantation (TAVI, *N* = 83), or surgical aortic valve replacement (AVR, *N* = 27). Overall, 22 patient-specific baseline parameters were tested for within-group prediction of resource use.

**Results:**

Baseline characteristics differed between groups and reflected the non-randomized, real-world allocation of treatment options. Overall procedural times were shortest for TAVI, intensive care unit (ICU) length of stay (LoS) was lowest for AVR. Length of total hospitalization since procedure (THsP) was lowest for TF-TAVI; 13.4 ± 11.4 days as compared to 15.7 ± 10.5 and 21.2 ± 15.4 days for AVR and TA-TAVI, respectively. For TAVI and AVR, EuroScore I remained the main predictor for prolonged THsP (*p* <0.01). Within the TAVI group, multivariate regression analyses showed that TA-TAVI was associated with a substantial increase in THsP (55 to 61 %, *p* <0.01). Additionally, preoperative aortic valve area (AVA) was identified as an independent predictor of prolonged THsP in TAVI patients, irrespective of risk scores (*p* <0.05).

**Conclusions:**

Our results demonstrate significant heterogeneity in patients baseline characteristics dependent on treatment and corresponding differences in resource utilization. Prolonged ThsP is not only predicted by risk scores but also by baseline AVA, which might be useful in stratifying TAVI patients.

**Trial registration:**

German Clinical Trial Register Nr. DRKS00000797

## Background

Due to the steadily increasing life expectancy in Western countries such as Germany, the prevalence of patients presenting with degenerative calcific aortic valve stenosis (AS) is rapidly increasing. As a result, both the clinical and economic aspects of their treatment are of high interest. The German Aortic Valve Registry (GARY) was initiated as a starting point for the investigation of these issues, and its first published findings confirm the desirability of better data on this subject [1–3].

Since the commercial launch of transcatheter aortic valve implantation (TAVI) in 2007, the technique has been widely adopted by cardiologists and cardiac surgeons, with more than 100,000 procedures performed worldwide by the end of 2013 [4, 5]. The results ofthe PARTNER trial have shown outstanding benefits over a period of 3 years in inoperable and high risk patients with severe AS [6–9]. However, for the technically operable high risk population surgical aortic valve replacement (AVR) continues to be regarded as the gold standard treatment due to the long-standing clinical experience with the technique. Despite this there is an ongoing debate, notably in the light of the recently updated guidelines on the management of valve disease from the ESC & EACTS, as to where these two therapeutic alternatives should be positioned from both the clinical, and, inevitably, economic perspectives [10].

A better resource utilization is becoming a “real” problem in the medical field. A correct evaluation of the costs has to be done for every new procedure.

Due to the technique’s relatively recent introduction into clinical practice, several analyses measuring the in-hospital costs and cost-effectiveness of TAVI have been published in the last two years, and these have been reviewed recently [11–15]. They all present their results for either inoperable (deemed unsafe for surgery) or high-risk patients (where surgery is not ruled out, but not without substantial risk), and overall TAVI is shown as cost-effective versus medical therapy/conservative treatment, however for TAVI versus surgical AVR the results are mixed. Although these studies aimed to make a comparison of the economic aspects of the treatments, they do not directly compare detailed resource usage.

This study sought to define and evaluate preoperative risk factors suitable for the prediction of comparative resource utilization in the treatment of symptomatic AS in elderly patients based on a real world patient cohort enrolled in the “TAVI calculation of costs trial” (TCCT).

## Methods

### Study design

The TAVI calculation of costs trial (TCCT) was designed as a prospective observational multicenter cohort study on elderly patients with symptomatic AS receiving either AVR or TAVI. This study was approved by the institutional ethics committee (Research Ethics Committee Albert-Ludwigs-Universität Freiburg, Germany, ID: 52/11), and was registred in the German Clinical Trial Register (ID: DRKS00000797). All patients referred between April 2011 and March 2013 to our centers were considered for inclusion into the study. Age above 75 years was deliberately chosen as inclusion criteria with the intention to avoid overwhelming the AVR treatment arm with data on substantially lower risk patients. All treatment decisions were made by a study-independent “heart team” of cardiac surgeons and cardiologists according to best clinical practice. A total of 10 AVR patients that underwent additional coronary artery bypass grafting were excluded from the dataset due to the existence of an additional procedure.

### Resource utilization

A primary focus of the study was the use of healthcare resources in treatment. To this end, we examined the complete duration of hospital length of stay (LoS) from admission until final discharge home or to a rehabilitation facility. We approached this by distinguishing two components: 1) procedure-to-discharge LoS; the time from procedure to discharge from the treating centre, generally corresponding to hospital LoS in the literature, and 2) post-discharge LoS; the time from initial treating hospital discharge to final discharge from any other subordinate hospital. From these parameters, we further derived a highly useful overall measure of LoS, the “total hospitalisation since procedure” (THsP); combining procedure-to-discharge LoS and post-discharge LoS. For death prior to discharge, LoS was defined as the time from procedure until the day of death. In addition, all further relevant resource utilization characterized by specific procedural parameters, e.g. duration on ventilator, was also recorded and analyzed.

### Statistical analysis

Quantitative continuous variables are described with means ± standard deviations, and quantitative discrete variables with absolute and relative frequencies. Differences between treatment groups were analysed using the non-parametric Wilcoxon rank-sum test or Fisher’s exact test. In order to identify suitable within group predictors for in-hospital resource utilization, all baseline parameters were tested against THsP, intensive care unit (ICU) LoS, and time on ventilator. Complete information about resource use can only be retrieved for individuals who are alive at follow-up, but a simple exclusion of individuals with missing information can lead to serious bias in risk prediction. Accordingly, patients who died during hospital stay were censored using right-censored normal regression analyses. In addition, the positive skew of the resource use variables wasaccounted for by application of the natural logarithm of all dependent variables (ln(x + 1)) in the few cases where time on ventilator or ICU LoS were equal to zero). Accordingly, the results may be interpreted as the relative increase of the outcome (percentage changes in the ‘Length of hospital stay’, the ‘Length of mechanical ventilation’ or the ‘Length of ICU stay’) when comparing the two levels of a binary covariate or a change by 1 unit of a continuous covariate. Mean conditional imputation was applied for missing variables. All analyses were performed using Stata 12 (StataCorp, College Station, TX, USA).

## Results

### Patient population

The total study population comprised 110 patients undergoing TF-TAVI (*n* = 60), TA-TAVI (*n* = 23), and AVR (*n* = 27).

Baseline demographics and characteristics are provided in Table 1. As treatment decisions were based on clinical judgement according to patient presentation and the decisions of a “heart team”, there were substantial differences between groups. AVR patients were significantly younger (*p* <0.01) and exhibited significantly lower EuroScore I (*p* <0.01), Euroscore II values (*p* <0.01) and STS-Scores (*p* <0.01) than the TAVI patients. Among TAVI patients, patients with a TA approach were, on average, at higher risk than TF-TAVI, although the differences in EuroScore I, EuroScore II and STS score values were not statistically significant (*p* = 0.065, *p* = 0.094, *p* = 0.505, respectively).

**Table 1:** Mean baseline characteristics of TAVI and AVR patients aged ≥ 75 years

	AVR		TAVI						AVR vs. TAVI	
			All		TF-TAVI		TA-TAVI			TF vs. TA-TAVI
	27			83	60		23		*p*-value	*p*-value
Demographics										
Age	78.74	±3.43	82.9	±4.48	82.95	±4.67	82.78	±4.03	*p* <0.01	0.963
Female	44 %		67 %		68 %		65 %		0.029	0.798
Medical History										
LVEF (%)	50.37	±12.8	50.52	±9.62	51.2	±9.25	48.47	±10.68	0.785	0.257
AVA	0.81	±0.17	0.68	±0.16	0.68	±0.17	0.65	±0.14	*p* <0.01	0.9796
Renal disease^a^	56 %		64 %		57 %		83 %		0.183	0.043
CAD	37 %		54 %		55 %		52 %		0.183	1.000
Previous MI	11 %		22 %		17 %		35 %		0.272	0.084
Previous PCI	19 %		24 %		23 %		26 %		0.609	0.781
Previous Stroke	4 %		7 %		5 %		13 %		1.000	0.340
Previous CABG	4 %		12 %		8 %		22 %		0.288	0.131
AF	33 %		41 %		33 %		61 %		0.507	0.027
Hypertension	93 %		83 %		85 %		78 %		0.348	0.518
DM	33 %		27 %		25 %		30 %		0.623	0.782
Liver disease	4 %		6 %		5 %		9 %		1.000	0.614
COPD	11 %		16 %		13 %		22 %		0.757	0.336
PVD	4 %		20 %		17 %		30 %		0.068	0.224
Quality of life^b^	0.84	±0.21	0.79	±0.21	0.8	±0.22	0.76	±0.17	0.062	0.134
proBNP (pg/ml)	1474	±1708	4098	±6576	3820	±6111	4805	±7749	0.654	0.760
Risk Scores										
EuroScore I	10.44	±6.21	20.66	±13.05	18.53	±10.16	25.87	±17.49	*p* <0.01	0.065
EuroScore II	3.54	±3.15	6.77	±5.59	5.83	±4.09	9.04	±7.84	*p* <0.01	0.094
STS-Score	3.48	±1.57	5.7	±3.36	5.48	±2.88	6.21	±4.35	*p* <0.01	0.505

Abbreviations: *LVEF* – left ventricular ejection fraction; *AVA* – preoperative aortic valve area; *CAD* – coronary artery disease; *MI* – myocardial infarction: *PCI* – percutaneous coronary intervention; *AF* – atrial fibrillation; *DM* – diabetes mellitus; *COPD* – chronic obstructive pulmonary disease; *PVD* – peripheral vascular disease; *proBNP*–B-type natiuretic peptide. Data are presented as n (%) or mean ± standard deviation

^a^Renal disease defined as (glomerular filtration rate <60 ml/h); ^b^Patients quality of life at baseline according to standardized EQ-5D questionnaire

There also was a gender imbalance, with a higher proportion of females undergoing TAVI (*p* = 0.029). This may, however, be explained by the fact that women exhibited significantly higher EuroScore I values (18.62 in comparison to 15.56, *p* = 0.019). In addition, proBNP (B-type natiuretic peptide) increased in line with EuroScore. Patients with prior CABG were observed in 12 % of TAVI cases and 4 % for AVR. Peripheral vascular disease (PVD) was most frequent in the TA-TAVI groups, 30 % versus 4 % and 17 % for AVR and TF-TAVI.

### Resource utilization

AVR and TAVI procedural parameters (timings and staff) and patient LoS are detailed in Table 2. Overall, time in the cathlab or OR was shortest for TAVI and substantially longer for AVR (*p* <0.01). Both TF and TA-TAVI averaged around two hours (122 and 142 min, respectively), whereas AVR averaged 218 min. Total time, defined as the time a patient was anesthetised, was similarly diverse between TAVI and AVR patients (*p* <0.01). On average, staff numbers required per procedure were: 3.5 physicians (including one trainee) and 2.7 nurses for TAVI, and 2.7 physicians, 2.0 nurses, and a perfusionist for AVR. Notably, in our centres patients undergoing TAVI are anesthetised in the OR before moving to the cathlab, which substantially increases total time.

**Table 2:** Procedural times, staff utilization, patient in-hospital length of stay and discharge behaviour of TAVI and AVR patients aged ≥ 75 years

	TF-TAVI (60)	TA-TAVI (23)	AVR (27)
Procedural resource utilization			
Time cathlab/OR (min.)	122.3 ± 40.5 (118.5)	142.3 ± 96.9 (109)	217.7 ± 48.8 (223)
Staff			
Physician	3.58 ± 0.59 (4)	3.17 ± 0.72 (3)	2.67 ± 0.62 (3)
Nurse	2.55 ± 0.59 (3)	2.91 ± 0.29 (3)	2.04 ± 0.19 (2)
Time anesthesia (min.)	238.3 ± 55.4 (245)	247.5 ± 80.4 (220)	327.9 ± 57.6 (325)
Staff			
Physician	1.0 ± 0.0 (1)	1.96 ± 0.21 (2)	1.93 ± 0.27 (2)
Nurse	1.02 ± 0.34 (1)	0.32 ± 0.02 (0.33)	0.33 ± 0.15 (0.33)
Perfusionists^a^	0 (0)	0 (0)	1.0 ± 0.0 (1)
Post-procedural resource utilization			
Time spent in ICU (hours)	105.0 ± 198.6 (62.7)	105.4 ± 100.4 (66.1)	89.0 ± 131.0 (47.2)
Time on ventilation (hours)	35.8 ± 110.7 (8.6)	35.7 ± 64.6 (9.2)	42.6 ± 115.8 (12.8)
Length of stay (days)			
Procedure to discharge	13.1 ± 11.3 (10)	13.0 ± 6.6 (10)	10.6 ± 4.6 (10)
Post-discharge LoS	0.3 ± 1.4 (0)	8.2 ± 13.6 (0)	5.1 ± 7.0 (4)
Total hospitalisation since procedure	13.4 ± 11.4 (10)	21.2 ± 15.4 (17)	15.7 ± 10.5 (14)
Discharge destination			
Home	46.7 %	13 %	0 %
Rehabilitation	40 %	26.1 %	44.4 %
Hospital	5 %	43.5 %	51.9 %
Nursing Home	3.3 %	0 %	0 %
In-hospital death	5 %	17.4 %	3.7 %

Data are presented as mean ± standard deviation and median in parentheses

^a^In our centers an extracorporeal life-support system and perfusionists are on stand-by for all TAVI procedures

Time spent in the ICU varied greatly and was shortest for AVR at 89 h (median 47.2 h). Interestingly, there was very little difference in average ICU stay between TF-and TA-TAVI at 105 h for both. The mean duration of mechanical respiratory support was similar across the groups, around 40 h (median range 8.6–12.8 h). Procedure to discharge LoS was lowest for AVR (10.6 days) and similar for TF-TAVI and TA-TAVI, with 13.1 and 13.0 days respectively. Notably, post-discharge LoS with TA-TAVI and AVR was 8.2 and 5.1 days respectively, whereas for TF-TAVI it was only 0.3 days. The large differences in the mean post-discharge LoS between the different groups is mostly explainable by the fact that TF-TAVI patients were less often discharged to another hospital than TA-TAVI and AVR patients. Average THsP by group, including time spent in subordinate hospitals, therefore was, 13.4 days for TF-TAVI, 15.7 days for AVR, and 21.2 days for TA-TAVI. Accordingly, THsP was different between TF and TA-TAVI patients (*p* <0.01), but relatively equal between AVR and TAVI patients (*p* = 0.3058).

#### Predictors of in-hospital resource use following TAVI

According to univariate regression analyses (see Table 3), significant predictors (*p* <0.05) for prolonged THsP were: TA procedure, aortic valve area (AVA), history of atrial fibrillation (AF) and/or PVD, as well as higher EuroScore I, EuroScore II and STS Score.

**Table 3:** Univariate predictors of in-hospital resource use following TAVI (*N* = 83)

	Length of THsP (in days)		Length of mechanical ventilation (in hours)		Length of ICU stay (in hours)	
	% change	*p*-value	% change	*p*-value	% change	*p*-value
TA	69.05 %***	(0.000)	65.04 %	(0.158)	4.97 %	(0.820)
Age (per one-year change)	0.81 %	(0.620)	4.57 %	(0.216)	1.64 %	(0.371)
Female	6.16 %	(0.669)	−25.17 %	(0.440)	−4.81 %	(0.813)
Quality of life	−1.06 %	(0.686)	−0.34 %	(0.959)	−1.62 %	(0.589)
LVEF (per one-unit increase)	−1.72 %	(0.085)	−4.60 %*	(0.041)	−2.18 %	(0.139)
AVA (for increase of 0.1 cm2)	−5.62 %*	(0.018)	−8.08 %*	(0.042)	−5.40 %	(0.069)
GFR (per one-unit change)	−0.58 %	(0.060)	−1.26 %	(0.062)	−0.77 %*	(0.031)
CAD	20.20 %	(0.158)	52.20 %	(0.194)	19.72 %	(0.290)
Previous MI	5.50 %	(0.762)	24.23 %	(0.580)	−1.34 %	(0.952)
Previous PCI	17.94 %	(0.305)	−9.61 %	(0.792)	1.21 %	(0.957)
Previous Stroke	46.67 %	(0.304)	29.30 %	(0.390)	31.00 %	(0.451)
Previous CABG	10.74 %	(0.546)	−15.38 %	(0.730)	−10.60 %	(0.673)
AF	34.31 %*	(0.042)	54.96 %	(0.205)	33.51 %	(0.130)
Hypertension	−24.87 %	(0.152)	−60.90 %*	(0.048)	−39.47 %*	(0.016)
DM	7.14 %	(0.668)	11.18 %	(0.797)	−0.63 %	(0.979)
Liver disease	33.78 %	(0.364)	−6.11 %	(0.953)	5.24 %	(0.908)
COPD	−2.25 %	(0.876)	116.19 %	(0.184)	49.18 %	(0.128)
PVD	46.37 %*	(0.021)	44.63 %	(0.441)	33.91 %	(0.282)
EuroScore I (per one-unit change)	1.37 %*	(0.010)	4.76 %***	(0.000)	1.89 %*	(0.014)
EuroScore II (per one-unit change)	2.76 %*	(0.039)	6.82 %	(0.064)	3.05 %	(0.158)
STS Score (per one-unit change)	3.71 %*	(0.032)	10.05 %	(0.109)	6.27 %	(0.068)
proBNP (per 100 pg/ml change)	0.03 %	(0.709)	0.14 %	(0.675)	0.20 %	(0.253)

*p*-values in parentheses; *p <0.05, **p <0.01, ***p <0.001

**Fig. 1:**
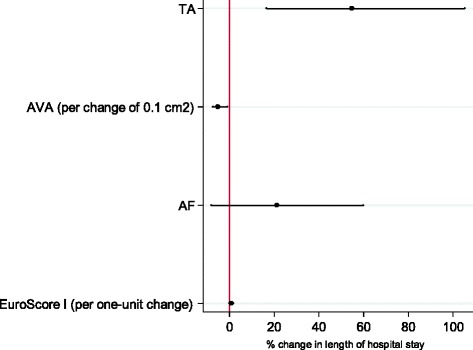
Multivariate predictors of Length of hospital stay following TAVI (*N* = 83). Point estimates and confidence intervals of the changes in length of hospital stay (see model (1) in Table 4)

The standard risk scores already cover most of the parameters identified in our calculation as predictive for an increased resource usage. However, neither AVA or AF are included in the cacluation of these scores, and so we hypothesised that they might represent additional variables which could be used by the clinician to predict outcomes. Accordingly, we fitted three multivariate regression models in order to analyse whether the baseline parameters of AVA and AF as well as the access route (TA) are predictors of in-hospital resource use for TAVI, independent of the predictive value of the three risk scores. As shown in Table 4, the TA approach was still associated with a substantial (approximately 55 to 61 %) increase in THsP. Moreover, AVA appeared to be an independent predictor of THsP irrespective of which risk-score we adjusted for: Table 4, model (1) contains an adjustment for the EuroScore I, model (2) for the EuroScore II and model (3) for the STS-Score. If we focus on AVA as a predictive factor additional to EuroScore I as shown in model (1), our results indicate that for every reduction in AVA of 0.1 cm^2^, the predicted THsP increases by 5.2 %, which is equivalent to almost one additional day of hospitalization (see also Fig. 1).

**Table 4:** Multivariate predictors of Length of hospital stay following TAVI (*N* = 83)

	Length of hospital stay (THsP)					
	model 1		model 2		model 3	
	% change	p-value	% change	p-value	% change	p-value
TA	54.65 %**	(0.003)	55.89 %**	(0.003)	61.45 %**	(0.001)
AVA (per change of 0.1 cm^2^)	−5.20 %*	(0.025)	−5.50 %*	(0.015)	−5.28 %*	(0.022)
AF	21.17 %	(0.171)	20.20 %	(0.197)	18.65 %	(0.241)
EuroScore I (per one-unit change)	0.83 %	(0.067)				
EuroScore II (per one-unit change)			1.42 %	(0.210)		
STS Score (per one-unit change)					1.83 %	(0.220)
*N*	83		83		83	

*p*-values in parentheses; *p <0.05, **p <0.01, ***p <0.001

#### Predictors of in-hospital resource use following AVR

Similarly, for AVR (see Table 5) a significant predictor in univariate analysis (*p* <0.05) for prolonged THsP were the STS-Scores. Moreover, a low value of the glomerular filtration rate at baseline was a predictor for prolonged time on ventilation, whereas patients with a medical history of diabetes spent less time in ICU settings.

**Table 5:** Univariate predictors of in-hospital resource use following AVR (*N* = 27)

	Length of THsP (in days)		Length of mechanical ventilation (in hours)		Length of ICU stay (in hours)	
	% change	p-value	% change	p-value	% change	p-value
Age (per one-year change)	0.55 %	(0.757)	1.63 %	(0.716)	3.87 %	(0.262)
Female	16.88 %	(0.382)	−33.77 %	(0.370)	−13.76 %	(0.662)
Quality of life^#^	−12.98 %	(0.648)	16.07 %	(0.850)	−3.93 %	(0.962)
LVEF (per one-unit change)	−0.34 %	(0.480)	0.05 %	(0.975)	0.60 %	(0.413)
AVA (per change of 0.1 cm2)	−43.45 %	(0.295)	−86.15 %	(0.134)	−41.02 %	(0.594)
GFR (per one-unit change)	−0.54 %	(0.271)	−2.55 %*	(0.050)	−0.82 %	(0.362)
CAD	−2.46 %	(0.903)	58.88 %	(0.342)	−2.38 %	(0.949)
Previous PCI	−8.61 %	(0.596)	−1.76 %	(0.954)	−17.88 %	(0.565)
AF	1.18 %	(0.943)	87.95 %	(0.157)	49.03 %	(0.203)
Hypertension	−14.53 %	(0.121)	26.49 %	(0.370)	10.45 %	(0.894)
DM	−1.62 %	(0.935)	−20.63 %	(0.549)	−49.49 %*	(0.015)
COPD	25.86 %	(0.480)	−34.82 %	(0.178)	−18.21 %	(0.373)
EuroScore I (per one-unit change)	1.71 %	(0.063)	1.93 %	(0.638)	3.11 %	(0.167)
EuroScore II (per one-unit change)	4.48 %	(0.086)	4.92 %	(0.605)	5.01 %	(0.257)
STS Score (per one-unit change)	9.48 %**	(0.007)	24.86 %	(0.117)	16.30 %	(0.133)
proBNP (per 100 pg/ml change)	0.045 %	(0.721)	0.104 %	(0.712)	0.011 %	(0.958)

*p*-values in parentheses; *p <0.05, **p <0.01, ***p <0.001

Not tested due to an insufficient number of events: previous MI, previous stroke, previous CABG, liver disease, PV

## Discussion

Our investigation showed that resource utilization differs substantially and significantly between therapy options for severe AS in the elderly, with TF patients experiencing shorter overall LoS (THsP) compared to other options. We hypothesise that the early diagnosis and treatment of symptomatic patients is optimal for reducing healthcare resource utilization and have shown that AVA, as a surrogate for the progress of the disease, is an independent predictor of this. As it is not considered in the three commonly used risk scores, we suggest that AVA can be an additional predictor of clinical and economic outcomes.

Recently, the national GARY registry has published the first data for TAVI and AVR across Germany. Broadly speaking our results are consistent with this data, certainly in terms of trends between treatments, although the specific measures that are reported in the paper do differ in magnitude. Although no data was given on THsP, the authors do comment in their discussion that “*The length of hospitalization was rather similar* [between the different treatment groups], *and accordingly no critical factor in comparing the techniques*”, which may be a point of difference between the two studies [3].

Hospital LoS is a metric that is highly relevant for local and national level decision makers due to its obvious impact on resources and operating logistics within secondary healthcare settings. Not only absolute values are important, but also variability and predictability, in order to improve efficiency. We report highly transparent the length of hospitalisation and show large differences and corresponding variability, with TF-AVI and AVR patients having the shortest LoS. Comparisons with other studies are inherently challenging due to a lack of uniform definitions, variations between healthcare systems, and limited data on alternative, heart-team established strategies. LoS generally ranges between 5 and 13 days for TAVI, [16–18] and 9 and 20 days for AVR [19–22]. But as with other studies, the LoS is not only a consequence of clinical outcomes but also local management practice and, potentially, funding considerations. Without a common definition of LoS that includes stays in other facilities immediately after discharge from the treating centre, it is difficult to compare and interpret the data. We suggest that length of “total hospitalisation since procedure” (THsP) is comprehensive.

As a result of the described risk-selection, comparisons between treatment groups or risk prediction may always be subject to a substantial selection bias. In contrast, within group risk prediction is more appropriate and, according to our univariate and multivariate analyses, TA procedure and AVA were significant and independent predictors of THsP irrespective of the common risk scores. It is well known that TA-TAVI has different outcomes compared with TF-TAVI, and this is due to multiple factors predominantly related to underlying differences which drive the choice of a TA approach rather than the TF route. AVA presents a new concept, which however is probably best interpreted as a marker for worsening medical condition. As the AVA decreases and the stenosis pathology worsens, the effects on the cardiac and circulatory systems, even at the sub-clinical level, lead to a poorer prognosis and the need for more resource utilization for patient recovery. As mentioned above, AVA is not used in the commen risk scores, but parameters associated with the development of degenerative aortic valve stenosis such as chronic kidney failure or patient age are. Thus, when caluclating the risk scores, AVA might have an indirect impact on the result of the risk scores.

Most previous studies focusing on risk prediction for TAVI and/or AVR focus on postoperative factors, such as complications, for predicting clinical and economic outcomes [15, 18, 22–29]. In one of the few exceptions, Green et al. analyzed the association between preoperative frailty status and LoS in elderly patients (mean age 86 years), and showed that a high frailty score was associated with a longer LoS [29]. We examined the impact of self-reported quality of life as this is the only part of this frailty score included in our study, but it didn’t show any impact on LoS.

There are multiple benefits to being able to preoperatively predict resource usage for patients. From a hospital perspective it allows the best placement of efforts and optimal planning to achieve the greatest throughput of patients whilst achieving the best clinical outcomes. This is obviously an asset for the hospital as it maximises productivity and limits losses. Efficient planning should enable more procedures whilst maintaining consistent staff levels and should result in fewer cancelled elective procedures as a consequence of unavailability of beds. This also has a direct benefit for the patient, who is more likely to receive optimal planned care and to experience minimal delays in procedures, transfers to other facilities, etc. All of these may be compromised by unexpected resource utilization leading to substandard care resulting from pressures on the system. Finally, the drive towards reducing THsP is also due to the benefit of reducing non-procedural secondary complications such as hospital acquired infections.

### Study limitations

Strikingly, within the boundaries of the study design and inclusion/exclusion criteria, there appears to be a number of important differences in patients from either the TAVI and the AVR group. Therefore, it would be highly biased and consequently misleading to make direct comparisons of the results between the groups. As shown in Table 2, outliers strongly affect the force of expression, particularly in small numbers of cases. In contrast to purely medical approaches, however, outliers are of particular interest in health economic considerations. From a societal perspective, outliers influence the overall costs of the treatment. In addition, outliers also affect the gap between actual costs and reimbursement from a hospital perspective and are therefore critical for a number of economic considerations from different perspectives.

Moreover, our initial study design included the enrollment of drug patients, analagous to the conservative treatment arm from PARTNER B, but it was soon evident that these patients were rarely considered inoperable and were typically offered TAVI. This directly reflects the current clinical practice in our, and presumably other centers, and demonstrates the very limited number of patients presenting with a “true” contraindication for TAVI or AVR. It however remains unclear whether these “borderline” patients are a consequence of a shift in perceptions about eligibility for TAVI or surgery, and if so what impact their inclusion will have on mortality and mobidity affecting comparisons between with pivotal studies such as PARTNER.

## Conclusion

We present here results from the prospective TAVI Calculation of Costs Trial (TCCT) aimed at evaluating the in-hospital resource use in the treatment of severe aortic valve stenosis in elderly patients. Our findings reveal fundamental differences between patient groups, and different requirements and use of resources. We show that, in addition to the commonly used risk scores, in TAVI patients the preoperative aortic valve area is an independent and so far unknown predictor of hospital length of stay.
